# Antimicrobial activity and carbohydrate metabolism in the bacterial metagenome of the soil-living invertebrate *Folsomia candida*

**DOI:** 10.1038/s41598-019-43828-w

**Published:** 2019-05-13

**Authors:** Valeria Agamennone, Ngoc Giang Le, Nico M. van Straalen, Abraham Brouwer, Dick Roelofs

**Affiliations:** 1 Department of Ecological Science, VU University Amsterdam, Amsterdam, The Netherlands; 2 Department of Microbiology and Systems Biology, TNO, Zeist, The Netherlands; 3 Institute of Biotechnology, Vietnam Academy of Science and Technology, Hanoi, Vietnam; 4 BioDetection Systems, Amsterdam, The Netherlands

**Keywords:** Metagenomics, Symbiosis, Metagenomics, Microbiome

## Abstract

The microbiome associated with an animal’s gut and other organs is considered an integral part of its ecological functions and adaptive capacity. To better understand how microbial communities influence activities and capacities of the host, we need more information on the functions that are encoded in a microbiome. Until now, the information about soil invertebrate microbiomes is mostly based on taxonomic characterization, achieved through culturing and amplicon sequencing. Using shotgun sequencing and various bioinformatics approaches we explored functions in the bacterial metagenome associated with the soil invertebrate *Folsomia candida*, an established model organism in soil ecology with a fully sequenced, high-quality genome assembly. Our metagenome analysis revealed a remarkable diversity of genes associated with antimicrobial activity and carbohydrate metabolism. The microbiome also contains several homologs to *F*. *candida* genes that were previously identified as candidates for horizontal gene transfer (HGT). We suggest that the carbohydrate- and antimicrobial-related functions encoded by *Folsomia*’s metagenome play a role in the digestion of recalcitrant soil-born polysaccharides and the defense against pathogens, thereby significantly contributing to the adaptation of these animals to life in the soil. Furthermore, the transfer of genes from the microbiome may constitute an important source of new functions for the springtail.

## Introduction

Microorganisms inhabit every type of environment, and many live in association with eukaryotic hosts. These microbes can influence their host’s ecology and evolution by contributing to a variety of processes such as digestion, immunity, and protection from pathogens^1^. Hexapods are good models to study host-associated microorganisms: they constitute the most diverse and abundant group of eukaryotic organisms on earth, and in many cases the establishment of specific microbial symbioses may have provided the key for their evolutionary success. Some hexapods depend on microbial symbionts for nutritional or defensive purposes^2,3^, suggesting that a good understanding of their biology should include the study of their associated microbes. This has been described as a “new imperative for the life sciences”^4^.

The majority of microorganisms is not accessible through traditional culturing techniques^5^ and metagenomic sequencing is an appropriate tool to study the diversity of species and functions of microbes in different ecosystems^6^. Metagenomics of insect-associated microbial communities has provided important insights in the interactions between microorganisms and their hosts, including the discovery of metabolites with potential biotechnological applications. For example, metagenomics of a termite’s gut microbiota has elucidated the mechanisms underlying wood degradation in this environment, while also identifying bacterial enzymes with interesting hydrolytic functions^7^. Other studies have found that microbial symbionts of insects are important sources of novel antimicrobials^8^.

The springtail *Folsomia candida* Willem 1902 (Hexapoda: Collembola) is a small invertebrate living in soil environments, where it feeds on fungal hyphae, decaying organic material and microorganisms. This species is a commonly used test organism in ecotoxicology and in ecogenomics^9^ and recently its genome and transcriptome have been sequenced^10^. Approximately 2.8% of the genes in the genome of *F*. *candida* are of foreign origin, having been acquired from bacteria and fungi through HGT^10^. Many of these genes are involved in carbohydrate metabolism, specifically in cell wall degradation; these functions may aid the animal in extracting nutrients from polysaccharides resulting from the degradation of plant and fungal biomass in the soil. In addition, several foreign genes are involved in antibiotic biosynthesis^11,12^. These genes are strongly induced by stress exposure^13,14^ and it is hypothesized that they may be involved in regulating the composition of gut microbial communities in *F*. *candida*^15^, or in protecting the springtails from pathogens. In fact, *F*. *candida* has been shown to be non-susceptible to some microbial pathogens present in soil environments^16,17^.

Recently, we have shown that bacteria isolated from this springtail display inhibitory activity against a variety of pathogens, including entomopathogenic soil fungi^18^. This suggests that the microbiota associated with *F*. *candida* may be a source of antimicrobial compounds, most likely involved in regulatory and defensive functions. Similar mechanisms have been observed in the honey bee: here, symbiotic lactic acid bacteria (LAB) active against transient environmental microbes are suggested to play an important role in the establishment and maintenance of a normal gut microbiota through the production of various antimicrobial agents^19^. Furthermore, the gut microbiota of *F*. *candida* may be involved in the breakdown of dietary component and in the uptake of nutrients. A nutritional role of gut microorganisms has been described for many other invertebrates and animals in general^1,20^. Even though the exact role of the gut microbiota in *F*. *candida* and its potential nutritional and defensive functions still need to be elucidated, we suggest that gut bacteria are an important factor interacting with the springtail, and that they provide physiological traits advantageous to thrive in a microbe-dominated environment such as the soil.

In this paper, we provide the first functional description of the gut bacterial community of a springtail based on a whole-metagenome sequencing approach. We hypothesize that the gut microbiome may aid in nutrient uptake and pathogen defense of *F*. *candida*^1^, thereby optimizing the fitness of the host. Furthermore, both functions are of potential interest for biobased applications: we identified a number of enzymes involved in lignocellulose break down and encoding compounds with predicted antimicrobial activity. Aside from constituting beneficial traits for an animal living in the soil environment, these functions may also represent good targets for drug discovery and for the development of biotechnological applications. Using a comparative analysis between genes of the gut microbiome and foreign genes in *F*. *candida*, we have identified functions possibly assimilated by the host through HGT.

## Results

### Sequencing results, assembly and annotation

Table 1 summarizes the sequencing results by indicating, for each sample, the preparation method used and the number of raw and filtered reads obtained. Approximately 90% of the reads passed the trimming step. Most of these reads (more than 97%) originated from the host *Folsomia candida*, and were removed during the next filtering step along with reads from *Wolbachia pipientis*, *Saccharomyces cerevisiae* (used as food source for *F*. *candida*, and therefore likely to contaminate the genomic libraries) and human DNA. The proportion of reads of prokaryotic origin was slightly higher in dissected gut samples compared to whole springtail samples (compare sample Fc3 to Fc4), and it was much higher in samples treated with the cell-separation method compared to untreated samples (compare sample Fc2 to Fc4, and Fc1 to Fc3). When combining dissection and cell-separation, the proportion of prokaryotic reads increased by a factor 5 (compare sample Fc1 to Fc4). The lowest proportion of *Wolbachia* was observed in the FC3 sample (untreated dissected guts).

**Table 1:** Preparation method and number of raw and filtered reads obtained for each sample.

Sample ID	Sample type	Sample preparation method	Raw reads	Reads after trimming	Reads after bowtie	Filtered reads
Fc1	Dissected guts (1 000)	Filter and DNA isolation	138 555 106	121 428 759 (87.6%)	3 605 008 (2.6%)	5 806 361 (1.23%)
Fc2	Whole springtails (300) and dissected guts (400)	Filter + Percoll and DNA isolation	133 586 006	116 187 374 (87%)	1 811 553 (1.36%)	
Fc3	Dissected guts (250)	Direct DNA isolation	103 864 717	93 503 412 (90%)	535 052 (0.52%)	
Fc4	Whole springtails (60)	Direct DNA isolation	94 686 416	84 746 773 (89.5%)	372 193 (0.39%)	

For each sample, the number of raw reads and the numbers of reads surviving each processing step is indicated. The percentages in bracket indicate the numbers of reads after each step relative to the number of raw reads.

5 806 361 high quality paired reads were used for assembly, which resulted in 107 138 contigs with a total length of 69 Mb (Table 2). Prodigal predicted 147 851 protein-coding sequences (CDSs), 133 594 of which were annotated in Swiss-Prot (Supplementary File 1). 132 657 genes (99%) were of bacterial origin, 665 genes were annotated as Eukaryota, 209 as viruses, 33 as Archaea, 30 as vectors or uncultured microorganisms and 14 257 were unassigned. Supplementary Fig. 1 shows the length distribution of the contigs. The 20 longest contigs (more than 100 000 bp each) were assigned either to *Pseudomonas* or *Microbacterium*.

**Table 2:** Results of assembly and annotation.

Number of contigs	107 138
Largest contig (bp)	1 306 495
Total length	69 108 988
N50	2 514
N75	853
L50	1 835
L75	10 181
GC%	60.2%
Gene count	147 851
Genes with function prediction	133 500

N50 = the size of the contig that, together with the larger contigs, contains 50% of the total metagenome length; N75 = the size of the contig that, together with the larger contigs, contains 75% of the total metagenome length; L50 = number of contigs whose summed length is 50% of the metagenome size; L75 = number of contigs whose summed length is 75% of the metagenome size.

### Taxonomic classification

**Figure 1:**
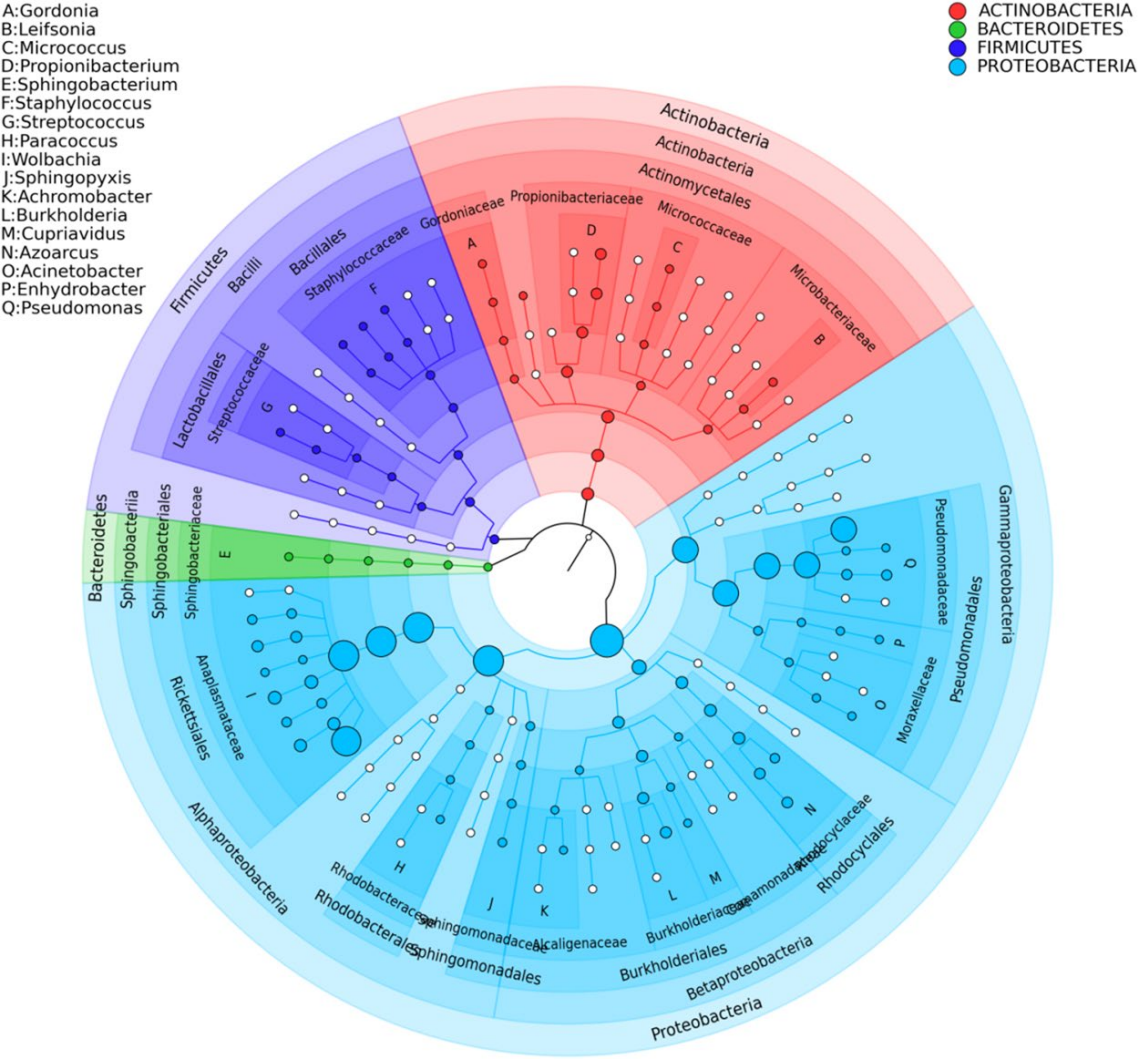
Phylogenetic distribution of the bacterial community in the metagenome of *F*. *candida*. The size of the circles is proportionate to the abundance of the taxa. The phylogeny was built based using Metaphlan on high quality raw reads.

The dominant bacterial taxa in the metagenome of *F*. *candida* were Proteobacteria (50% of the reads), Actinobacteria (32%), Bacteroidetes (12%) and Firmicutes (6%) (Fig. 1). These phyla constituted 99.5% of all the reads. 35 additional phyla were found in the remaining 0,5% of reads. 826 bacterial genera (excluding singletons) were identified. 23 of these genera covered 83% of the reads. The most abundant genus was *Microbacterium* (Actinobacteria, 13.1% of the reads), followed by *Paraburkholderia* (Betaproteobacteria, 7.2%), *Pseudomonas* (Gammaproteobacteria, 6.3%), *Staphylococcus* (Firmicutes, 5.6%), *Sphingopixis* (Alphaproteobacteria, 5.5%), *Stenotrophomonas* (Gammaproteobacteria, 5.4%), *Pseudoxanthomonas* (Gammaproteobacteria, 5.4%), *Gordonia* (Actinobacteria, 4.1%), *Burkholderia* (Betaproteobacteria, 3.4%) and 14 other genera each with a relative abundance higher than 1%. The overview of the identified taxonomic groups at the phylum, class and genus level is give in Supplementary Fig. 2.

### Overall functional analysis

**Figure 2:**
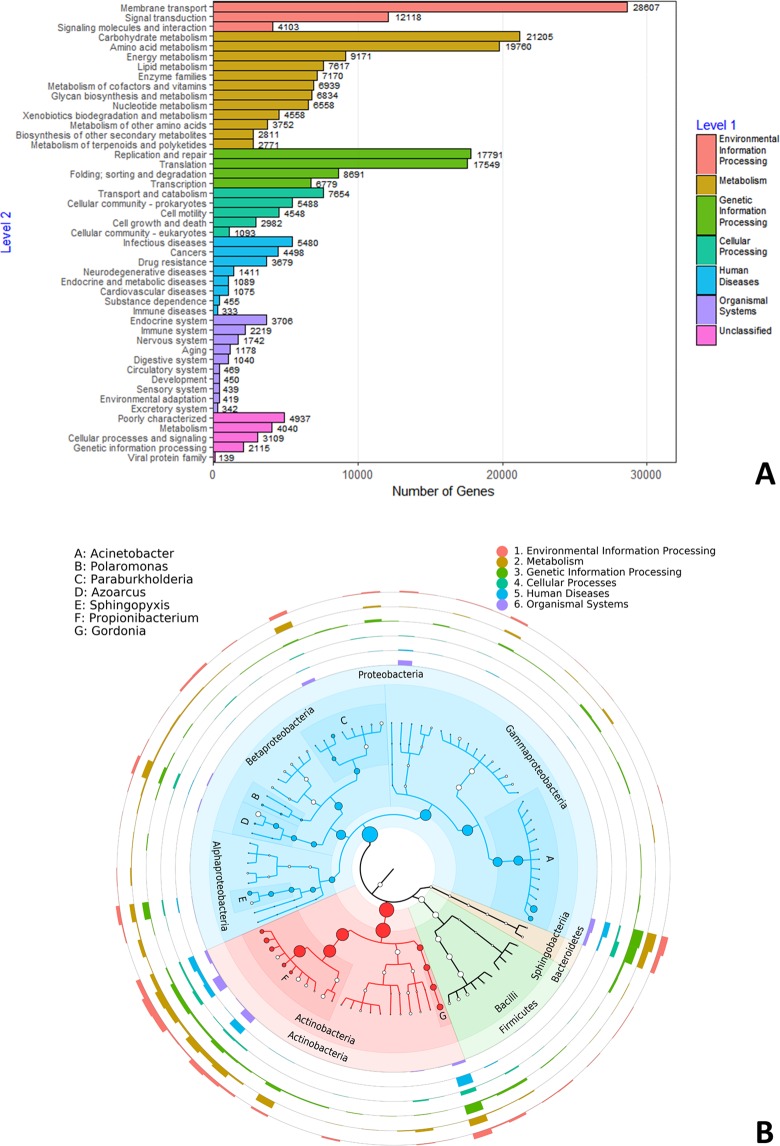
Functional annotation. (**A**) Detailed representation of the functional classes belonging to six main functional categories. (**B**) Functions mapped on the phylogenetic tree. The heights of the bars represent the numbers of kegg terms found for each bacterial species and for each functional category, in proportion to the width of the rings surrounding the taxonomic tree. A bar as high as the ring represents 50 kegg terms.

Comparison of the genes with the KEGG database recovered a number of functions. The most abundant functional categories were associated with membrane transport, signal transduction, carbohydrate and amino-acid metabolism, and the genetic information processes replication and repair and translation (Fig. 2A). Mapping of the functions on the phylogenetic tree shows that most predicted genes within any functional category are assigned to few bacterial species, namely the Proteobacteria *Acinetobacter johnsonii*, *A*. *lwoffii*, *Pseudomonas stutzeri*, *Paraburkholderia phytofirmans*, *Azoarcus toluclasticus*, *Sphingopixis alaskensis*, the Actinobacteria *Gordonia araii*, *Cutibacterium acnes* and three *Propionibacterium* species, and the Firmicutes *Staphylococcus equorum* (Fig. 2B). The next sections present the functions related to carbohydrate metabolism, secondary metabolite production and antibiotic resistance identified in *F*. *candida*’s microbiome.

### Carbohydrate metabolism

**Figure 3:**
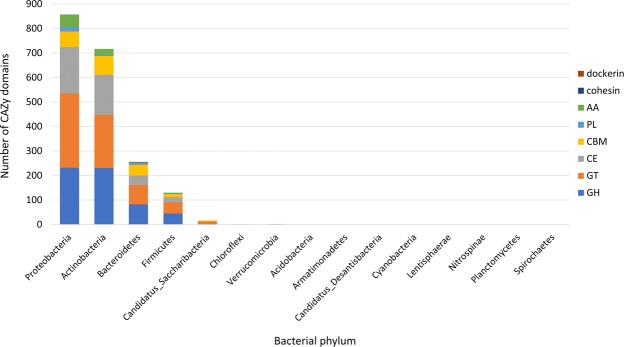
Column chart indicating the distribution of Carbohydrate Active EnZyme (CAZy) domains among the bacterial phyla retrieved in the metagenome. CBM: carbohydrate-binding module; CE: carbohydrate esterase; GH: glycoside hydrolase; GT: glycosyltransferase; AA: auxiliary activity; PL: polysaccharide lyase.

Carbohydrate metabolism was investigated by comparing predicted genes in *F*. *candida*’s microbiome with the carbohydrate-active enzymes (CAZY) database. 2 004 genes were predicted to code for enzymes involved in carbohydrate metabolism. 1 988 (99.2%) of these genes were of bacterial origin and they mostly originated from Proteobacteria (43%) and Actinobacteria (36%). The complete list of CAZymes is presented in Supplementary File 2, and an overview of the identified pathways involved in starch and sucrose metabolism is given in Supplementary Fig. 3. The carbohydrate-related genes were assigned to five CAZy classes and three modules (Fig. 3). 664 genes were identified as glycosyltransferases (GT, 33.1% of the total), 598 as glycoside hydrolases (GH, 30%), 420 as carbohydrate esterases (CE, 21%) and 206 as carbohydrate-binding modules (CBM, 10.1%). The GT, GH and CE CAZymes classes were overrepresented in the metagenome compared to the genome of *F*. *candida* (data not shown). Instead, enzymes with a carbohydrate-binding module (CBM) were more abundant in the genome of the host. 23 of the genes encoding carbohydrate-active enzymes had a best reciprocal blast hit against foreign genes in the genome of *F*. *candida*.

### Secondary metabolites

We screened the gut microbiome for the presence of secondary metabolite biosynthesis pathways related to antimicrobial activity. In total, 166 pathways were identified, 96 of which are putatively involved in the production of an unknown type of secondary metabolite (Supplementary Table 1). 32 pathways are related to saccharide or fatty acid-containing metabolites, and one cluster showed similarity to metabolites with both a saccharide and fatty acid component. Thirteen clusters are represented by non-ribosomal protein synthases (NRPS), which encode multi-domain and multifunctional enzymes involved in the biosynthesis of a large class of biologically active natural products. Another group of ribosomally-synthesized antimicrobial peptides, bacteriocins, are represented by four biosynthetic clusters. We also identified known antibiotics classes among the antismash clusters, namely rifamycin, spectinomycin, chalcomycin, and the antifungal bacillomycin.

### Antibiotic resistance

**Figure 4:**
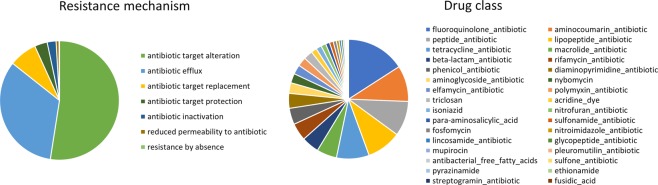
Overview of the drug mechanisms (left) and classes (right) associated with antibiotic resistance recovered in the metagenome of *F*. *candida*. The data was obtained by mapping predicted genes against the CARD database^21^ and by extracting the “resistance mechanism” and “drug class” categories from the results.

**Figure 5:**
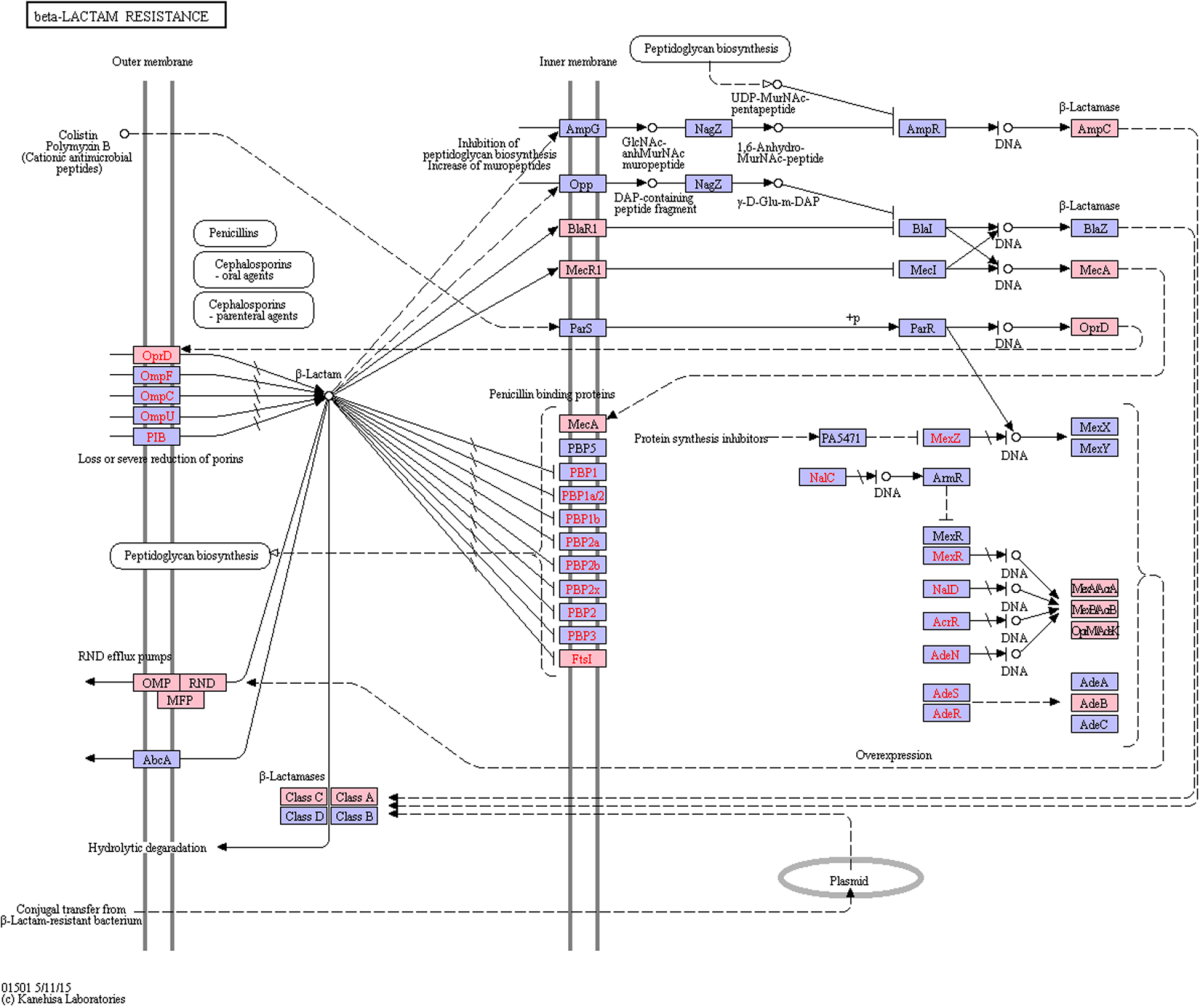
Genes from the *F*. *candida*’s metagenome predicted to be involved in β-lactam resistance are represented as colored items in KEGG’s β-lactam resistance pathway. The pathway map was obtained from the KEGG database^100^.

Predicted genes were mapped against the CARD database to determine the occurrence of antibiotic resistance genes (ARGs) in the gut microbiome of *F*. *candida*^21^. The analysis recovered 811 genes, corresponding to 209 unique terms in the CARD database. Figure 4 provides an overview of the identified antibiotic resistance mechanisms and of the drug classes to which resistance is conferred. The complete list of genes with accession and classification in CARD is provided in Supplementary File 4. Most antibiotic resistance mechanisms retrieved involved antibiotic target alteration (52%), followed by efflux processes (33%) and antibiotic target replacement (8%). The most abundant class of antibiotics associated with resistance was that of fluoroquinolones (16%), followed by aminocoumarins (10%), peptide antibiotics, lipopeptide antibiotics and tetracyclines (9% each), macrolides, beta-lactams and rifamycin (5% each). Several classes of ARGs involved in resistance to clinically relevant antibiotics, such as β-lactams and tetracycline, were identified (Figs 4 and 5).

### Host-microbiome interaction and horizontal gene transfer

A reciprocal blast was performed between the proteins in the *F*. *candida* genome and the predicted protein sequences in the metagenome, to identify orthologies between the springtails’ genome and metagenome. The list of best reciprocal blast hits was then compared with the list of 809 horizontally transferred genes in the genome of *F*. candida. We hypothesize that the identified orthologs between gut microbiome and the host genome have undergone HGT from the gut microbiome into the host genome.

**Figure 6:**
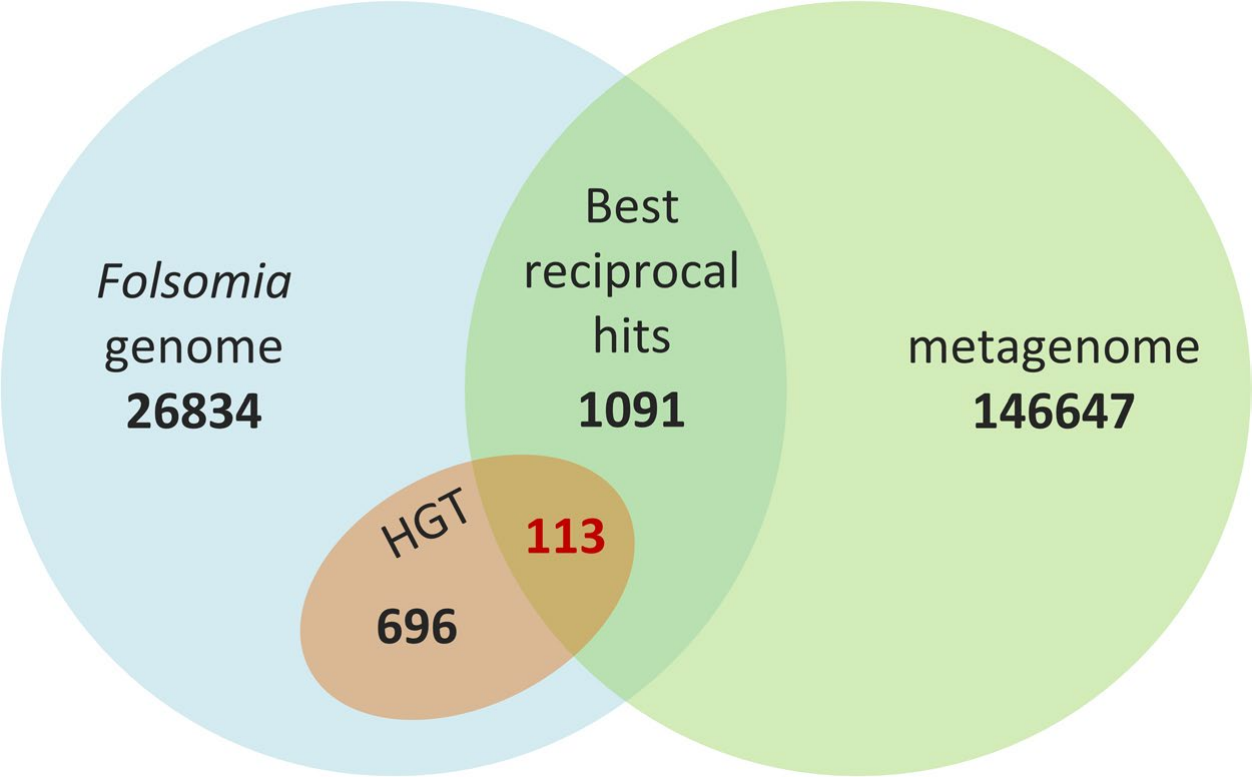
Venn diagram showing overlap (best-reciprocal blast hits) between proteins from *F*. *candida*’s genome (light-blue) and proteins from its gut microbiome (green). The red circle contains the horizontally transferred genes, and the number in red indicates the overlap with the gut microbiome.

Within the gut microbiome, 1 204 predicted protein sequences showed a best reciprocal blast hit with predicted protein sequences in the host genome. Most of these genes are involved in basic metabolic functions that are highly conserved across most life forms, such as transcription, translation, fatty acid metabolism, chaperone activity, amino acid biosynthesis, nucleic acid biosynthesis and ATP biosynthesis. Of these 1 204 genes, 113 had a best reciprocal blast hit against one of the 809 foreign genes in *F*. *candida* (Fig. 6). The complete list of these 113 genes is given in Supplementary File 5. Taxonomic and functional annotation suggests that *Pseudomonas*, *Microbacterium* and *Gordonia* may be the potential donors of 26, 12 and 9 genes respectively, jointly accounting for almost 50% of them (Supplementary File 5). Annotation analysis showed that 23 of the 113 genes are CAZymes. Supplementary Fig. 4 shows the predicted protein structures of both the metagenomic read and the animal contig for the top three reciprocal blast hits, corresponding to a glycosidase, an arabinosidase, and an isocitrate lyase. We also identified a non-ribosomal peptide synthase potentially involved in bacteriocin synthesis, one polyketide synthase and several enzymes associated with detoxification (monooxygenases ABC transporters, gluthatione-S-transferases, and copper oxidase). Most of the 71 remaining annotated genes are related to basic metabolic processes. Because we did not conduct gene expression analysis on the gut microbiome, we are currently unable to verify whether these genes are transcribed and thus functional in the microbial community.

## Discussion

In this study, we applied both dissection and a cell separation method to enrich the bacterial fraction of springtail samples, with the aim of increasing the proportion of bacterial reads after sequencing. The cell separation method was developed by Engel *et al*.^22^ and it was more effective than dissection when applied to *F*. *candida*. Although dissection normally helps to effectively target the microbial component^23^, this may be more complicated in microarthropods such as springtails because of their small size. A combination of dissection and cell separation method proved to be most effective in increasing the proportion of prokaryotic reads. Still, more than 97% of the reads in any sample belonged to the host *Folsomia candida*: recovery of genetic material from symbiotic microorganisms can be problematic in microhabitats such as insect guts, due to the much higher abundance of host DNA^24^.

*Wolbachia* can dominate the bacterial population in *F*. *candida*^25^. By discarding organs containing high amounts of *Wolbachia* (brain and ovaries), dissection should be effective in reducing the occurrence of the endosymbiont in the samples. Indeed, sample FC3 (consisting of guts obtained through dissection) had the lowest proportion of *Wolbachia* reads. Cell separation is also expected to reduce the amount of *Wolbachia* DNA in the samples. Because of its intracellular location (gut epithelium, ovaries and brain), a method that separates the eukaryotic cells from the prokaryotic ones without lysing them should be effective in reducing the amount of host and *Wolbachia* DNA in the same step. However, in this study, a combination of dissection and filtering resulted in an increased amount of *Wolbachia* reads (9.26% in sample FC1 vs 3.03% in sample FC2). Because of the difference in size between prokaryotic genomes and the host genome (resulting in sequencing biases), and because of possible lysis of host cells during the treatment of samples FC1 and FC2, resulting in the release of *Wolbachia* cells, it is difficult to conclude whether filtering was an effective strategy to reduce the representation of the endosymbiont in the metagenomic dataset.

The number of contigs and the total length after assembly are comparable to other soil invertebrate-associated metagenomes^26–28^. Although this was not attempted here, it may be possible to recover the genome of one or more species using the data collected in this study^29^.

With 826 bacterial genera identified, the level of diversity in *F*. *candida* approaches that described in the hindgut of termites, wood-feeding insects that have one of the most complex microbiota of any animal group^30^. Other soil invertebrates are characterized by comparable or even higher levels of microbial diversity. For example, Pass *et al*.^31^, studied the microbiome of the earthworm *Lumbricus rubellus* and found no less than 9 120 host-specific OTUs. This very diverse community was dominated by Proteobacteria and Actinobacteria, very similar to the situation in *F*. *candida*. High diversity was also observed in the gut of two cockroach species, with approximately 1 000 OTUs^32^, whereas slightly lower counts were detected in the ant *Cephalotes varians* (445 OTUs), in the compost worm *Eisenia fetida* (338 OTUs) and in the isopod *Armadillidium vulgare* (153 OTUs)^33–35^.

The bacterial community in *F*. *candida* was dominated by Proteobacteria species, and within this group the Gammaproteobacteria were particularly abundant (21% of the reads). Proteobacteria, a large taxon of functionally diverse bacteria, dominate the microbiome of terrestrial insects and other soil invertebrates such as earthworms, nematodes and isopods^31,36–39^. *Pseudomonas*, one of the most abundant bacteria detected in *F*. *candida*, is commonly found in the microbiome of soil invertebrates like termites, ants and beetles, isopods and nematodes, as well as in their environment^34,35,37,40^. *Pseudomonas*, together with *Rickettsia* and *Chryseobacterium*, was also the most abundant OTU in the microbiome of the springtail *Orchesella cincta*^41^. Another abundant bacterium in *F*. *candida* was *Paraburkholderia*. This genus includes many soil species, a few of which are used as plant probiotics thanks to their growth-promoting and possibly defensive properties^42^. Other members of the Proteobacteria identified in *F*. *candida*’s microbiota were *Sphingopixis*, *Stenotrophomonas*, *Pseudoxanthomonas*, *Burkholderia*, all of which were detected in soil invertebrates (worms, cockroaches, termites, ants and beetles)^37^. The most abundant bacterium in *F*. *candida* was *Microbacterium*. Members of the Microbacteriaceae have been previously identified in different species of beetles^43,44^, and Actinobacteria in general (although in low amounts) have been found in cockroaches^23^ and in a few species of insects (ants, beetles and termites) characterized by nutritional symbioses with fungi^33,40^. Actinobacteria are also one of the dominant bacterial groups in other soil invertebrates such as earthworms^31,34,45^.

The observed bacterial diversity in *F*. *candida* is comparable to that previously detected by 16S high-throughput sequencing in the same lab-reared population of springtails^25^. However, the taxonomic distribution between the two studies is very different. Based on 16S sequencing, *Pseudomonas* was the most abundant bacterial genus with 42% of the reads^25^. Nine other dominant OTUs were identified, including *Bacillus* (19% of the reads), a member of the Actinomycetales (9%), *Escherichia sp*. (4%) and *Ochrobactrum sp*. (3%). *Microbacterium* accounted only for 0.3% of the read, and *Paraburkholderia* was not identified. This discrepancy can be explained by the difference in sequencing methods applied. High-throughput amplicon sequencing is subjected to PCR bias, with differences in the amplification efficiency of DNA from different bacterial species; in shotgun metagenomic sequencing, on the other hand, biases can be caused by the method chosen for taxonomic assignment, possibly leading to misidentifications^46^.

The majority of reads in *F*. *candida*’s metagenome originated from pathways involved in membrane transport, carbohydrate and amino acid metabolism, replication, translation and repair. The abundance of genes involved in carbohydrate and amino acid metabolism may suggest a nutritional role of the microbiota. The springtails used in this study were reared exclusively on baker’s yeast (*Saccharomyces cerevisiae*), and specific microbial enzymes could aid in the breakdown of components of the fungal cell wall, including various polysaccharides and glycoproteins^47^. Natural populations of springtails may also benefit from the presence of such functions in their microbiome. In fact, carbohydrate-related functions are often enriched in the gut microbiome of different soil invertebrates, such as beetles, nematodes and isopods^27,39,44,48,49^, some of which rely on symbiotic microbes for the breakdown of long polymers such as lignin, cellulose and other plant-derived products^27,49^. *F*. *candida* is an euedaphic springtail species whose natural diet includes not only yeasts and other fungi, commonly occurring in the soil environment, but also decaying plant material. Recently, the microbiota of another springtail species, the epiedaphic *Orchesella cincta*, was studied, and some of the main functions predicted based on the microbial community structure were related to the breakdown of dietary components and of plant secondary metabolites^41^. In a previous study we observed substantial overlap in the composition of the bacterial communities between a lab-reared and a field population of springtails^25^. This suggests that similar carbohydrate-degrading functions may be present in both lab-reared and field populations of springtails.

Amino acid-related functions may also be beneficial for the host. Some intracellular endosymbionts biosynthesize essential amino acids that are lacking in the diet of their host^2^ and gut bacteria may exert similar functions^50^. A contribution to the host’s nutrition may also explain the abundance of functions related to membrane transport in *F*. *candida*. Transport allows host-symbiont exchanges and therefore it constitutes one of the most important functions in the maintenance of the symbiosis with bacteria providing nutrients^51^.

In accordance with the taxonomic assignment, most genes in the above discussed categories were predicted to belong to Proteobacteria and Actinobacteria species. Many genes were annotated to *Acinetobacter johnsonii*, a member of the Gammaproteobacteria that has been described as an opportunistic pathogens for animals as well as a possible reservoir of antibiotic resistance genes^52,53^. *Acinetobacter* was also a dominant genus in the microbiome of the earthworm *Eisenia fetida*^34^ and it was identified in other soil invertebrates such as the *Longitarsus* beetle and the isopod *Armadillidium vulgare*^35,43^. Many functions were also assigned to the genus *Propionibacterium*. This group of Actinobacteria includes species with good probiotic potential due to their capacity to modulate microbiota, gut metabolic activity and the immune system^54^. Interestingly, the immunomodulatory and anti-inflammatory properties of *Propionibacterium* have been observed not only in human and mouse models^54^, but also in soil invertebrates^55^. An abundance of genes was taxonomically assigned to a few other groups, among which *Gordonia*, a genus of Actinomycetes including many symbionts of terrestrial invertebrates^56^ and *Pseudomonas*, commonly found in soils and in soil invertebrates^37^.

Carbohydrate-degrading enzymes are commonly found in the bovine rumen^57^, in the gut of wood-feeding insects such as termites and woodwasps^7,58^ and in the microbial community of fungus gardens associated with leaf-cutter ants^59^. These enzymes are often of microbial origin, suggesting that herbivorous animals can exploit the catalytic activities of microbial symbionts to access nutrients stored in plant biomass^26^. In termites, the symbiotic relationship with a complex community of bacteria, archaea and protists in the gut enables the digestion of lignocellulose, conferring these insects a unique ecological position in tropical and subtropical ecosystems^49^. Whether similar relationships between Collembola and their microbiome exist is unknown at the moment, but microbial functions related to carbohydrate metabolism are likely to significantly contribute to the ecological role of springtails as members of the soil decomposer community.

Warnecke *et al*. found 700 glycoside hydrolase (GH) catalytic domains corresponding to 45 CAZY families in the microbiome of wood-feeding termites^7^. In the microbiome of *F*. *candida*, we identified a comparable number of genes encoding for enzymes with a capacity to break down long chain carbohydrates such as starch, lignin and cellulose. In nature, these enzymes may aid *F*. *candida* in extracting nutrients from the plant biomass that constitutes part of its diet, as was suggested for the springtail *O*. *cincta*^41^.

A large number of glycoside hydrolases was also observed among *F*. *candida* foreign genes^10^. Interestingly, some of the foreign genes that were also best reciprocal hits between the genome and the metagenome of *F*. *candida* were identified as CAZymes (Supplementary File 2). HGT of cellulose-degrading enzymes has been previously observed in plant-feeding insects^60^ and may be an important mechanism providing soil invertebrates with advantageous traits for living in the soil^61^.

The microbiome of *F*. *candida* contained several pathways responsible for the biosynthesis of secondary metabolites. This is a class of compounds that are often involved in competition and interaction between species, and they may contribute to the establishment and the maintenance of a stable gut microbiota through the exclusion of transient or pathogenic microbes^62,63^. Secondary metabolites often find applications in the biotechnological and medical sector. The main contributors to the identified pathways seem to be *Gordonia*, *Pseudomonas fluorescens*, *Bacillus* and *Streptomyces*.

A few of the identified pathways were represented by NRPSs, a class of enzymes responsible for the biosynthesis of natural products with a broad range of biological activities and pharmaceutical properties. Cluster 10 and 28 show resemblance with an NRPS producing pyoverdines, siderophores well known for their high affinity for Fe^3+^ under low iron availability^64^. Another NRPS involved in the biosynthesis of the siderophore nocobactin was identified in cluster 95. Three clusters show homology to NRPSs involved in antibacterial and antifungal activity. Cluster 31 shows substantial similarity (47%) to an NRPS producing orfamide, a compound of bacterial origin with antifungal properties and with good potential as biocontrol agent against fungal pathogens^65^. Cluster 130 represents an NRPS involved in microsclerodermin biosynthesis, an antifungal compound produced by a marine sponge^66^. A recent study also showed that this compound has properties of pharmaceutical relevance, as it can inhibit NFkappaB transcription in a human pancreatic cell line leading to apoptosis^67^. Finally, the NRPS identified in cluster 48 showed similarity to the NRPS involved in biosynthesis of the antibiotic caryoynencin, a compound originally isolated from a plant pathogen. Very recently it has been shown that this compound is produced by a symbiont of a herbivorous beetle, protecting its eggs against detrimental microbes^68^.

We also identified a number of bacteriocins, a class of compounds with potential as natural food preservative^69^. Many bacteriocins are biosynthesized by lactic acid bacteria, and in *Folsomia*’s gut microbiome these clusters are homologous to *Pseudomonas fluorescens* and *Gordonia effusa*.

Several other interesting biosynthesis clusters with functions related to medical applications were found, such as lymphostin, a known immunosuppressant isolated from *Streptomyces*^70^, and chartreusin, that exerts strong chemotherapeutic activity against various tumor cell lines^71^. We also identified a mangotoxin biosynthesis cluster. Mangotoxin causes apical necrosis of plant tissue, which may aid in food processing and digestion by the host^72^. Biosynthesis of the volatile compound homoserine lactone (hserlactone) may be related to communication between fungi and bacteria^73^, while ectoine may serve as osmolyte conferring resistance to salt, dessication and temperature stress^74^.

The distribution of antibiotic resistance genes (ARGs) in microbiomes sampled across environments and organisms is still not well understood. A large-scale metagenomics study indicated that soils harbor most classes of ARGs^75^. In the gut microbiome of *F*. *candida*, we identified over 200 unique terms associated with antibiotic resistance distributed over more than 800 genes, more than twice the number detected in human microbiomes and almost eight times the number detected in the giant African snail *Achatina*^76^. This might be explained by the intimate association between the springtail and the soil ecosystem.

The presence of ARGs in the gut of *Folsomia* may have ecological relevance. It is noteworthy that we identified a substantial number of β-lactamases, probably resulting from the selective pressure caused by β -lactam production by the host itself^12^. For example, *Bacillus toyonensis*, a member of *F*. *candida*’s microbiota, is highly resistant to β-lactams^77^. Furthermore, interactions between bacterial communities with antibiotic biosynthesis capacity and communities showing resistance to such antibiotics can also be expected. Observations from this and other studies indicate a potential for *Pseudomonas*, *Streptomyces* and *Gordonia* strains isolated from *F*. *candida* to synthesize antibiotics (see section above, Supplementary File 3 and^18^), while *Streptomyces*, *Enterococcus* and *Staphylococcus* are abundant among ARG-containing bacterial strains in *Folsomia*’s gut (Supplementary File 4). This supports the notion that antibiotics regulate the homeostasis of microbial communities, and may even be beneficial for commensal bacteria in environments such as the animal gut^78^. Finally, Engel & Moran^1^ suggested that this balance may be important in facilitating colonization resistance against parasites and bacteria pathogenic to the host. The data provided in this study will be highly relevant in formulating concrete hypotheses to investigate the ecological connectivity of antibiotic-biosynthetic and ARG-containing bacteria in gut microbiomes.

A previous study had identified 809 foreign genes in *F*. *candida*’s genome, which were validated by physical linkage with native genes (through PacBio long read single molecule sequencing), blast analysis and phylogenetic inference^10^. Moreover, Faddeeva *et al*. used RNA sequencing to show that almost 60% of this gene set was actively transcribed, indicating functional relevance^10^. Here, we applied best reciprocal blast analysis to identify microbial protein sequences orthologous to predicted protein-coding sequences in the genome of *F*. *candida*. We hypothesize that this would provide circumstantial evidence of horizontal gene transfer from members of the gut microbiome into the host genome. Indeed, within the gut microbiome we identified 113 best reciprocal blast hits with predicted protein sequences of foreign genes of the springtail, possibly indicating HGT from the gut microbiome to the host genome. The foreign genes without a best reciprocal blast hit within the gut microbiome may have been transferred from other microbial sources, for example the over 30% of foreign genes that conferred top blast hits with fungal donors^10^. Alternatively, other genes may have been transferred to the host genome early in the evolution of *F*. *candida*. In that case, the accumulation of mutations over time would lead to low similarity with members of the microbiome, preventing the identification of the possible source of these genes through best reciprocal blast searches. A number of foreign genes with best reciprocal blast hit with genes in the microbiome were CAZymes, involved in the degradation of polymers such as cell wall components. Gene transfer of carbohydrate-active enzymes may optimize the capacity of *F*. *candida* to extract nutrients from their diet^79^, thereby contributing to their adaptation to life in the soil.

Horizontal gene transfer from prokaryotes to eukaryotic host genomes has become a highly controversial topic. There are claims that gene transfer only occurs between hosts and mitochondria, plastids and endosymbionts, and that other HGT cases are the result of differential loss of ancestral genes, that originated prior to the last eukaryotic common ancestor^80^. However, this hypothesis overestimates gene contents of ancestral genomes, and is therefore unlikely^81^. We suggest that the foreign genes in *Folsomia*’s genome are most likely acquired via horizontal gene transfer^10^. Here, we propose that part of these HGT events could have taken place by interaction with the gut microbiota. In the gut environment host and microorganisms maintain an intimate physical association with many opportunities for interaction, thus increasing chances for gene transfer to occur^82^. Two recent studies provide evidence for bacterial DNA transfer into somatic human cells^83,84^ through bacterial type IV secretion system (T4SS). This system is known to mediate interbacterial conjugative DNA transfer and transkingdom protein transfer into eukaryotic host cells during bacterial pathogenesis. Schroder *et al*. showed that T4SS-dependent DNA transfer into host cells may occur naturally during human infection with *Bartonella*^84^. Furthermore, Ridley *et al*. identified a *Pseudomonas* strain as a donor of foreign DNA detected in human stomach carcinomas^83^. It is still unclear why functions that can be provided by the microbiome would be incorporated and maintained in *F*. *candida*’s genome. In the case of foreign genes involved in lignocellulose breakdown, we speculate that such functions, when controlled by the host, could provide fitness advantage in terms of energy balance and nutrient acquisition. Similarly, transferred genes involved in detoxification may protect the host for natural toxins that are quite common in the soil. These and other hypotheses should be tested by conducting gene knockdown and other experiments.

We have provided an insight in the metagenome of a collembolan species, *F*. *candida*. Most bacterial diversity is attributed to four phyla, that are also representative for soil microbial ecosystems, possibly confirming the interaction of *F*. *candida* with its natural environment. A broad spectrum of gene functions was identified, most notably related to carbohydrate metabolism, antibiotic resistance and secondary metabolite production. These functions were presented and discussed in the context of their ecological relevance and in the light of potential biotechnological applications. Finally, we presented data suggesting that the gut microbiome may have been a source of genes acquired by the host through HGT. These genes may have conferred a fitness advantage to the springtail, during adaptive evolution in the soil ecosystem.

## Materials and Methods

### Test organism

*Folsomia candida* individuals originated from a laboratory stock culture (“Berlin strain” VU University Amsterdam) that was originally established from specimens sampled in the field, and then maintained in stable laboratory conditions for several years. Springtails were cultured in plastic boxes with a bottom of plaster of Paris and charcoal. Cultures were kept in climate rooms at 20 °C temperature, 75% humidity and a 12 hour light-dark cycle. The springtails were fed dry baker’s yeast (Dr. Oetker, Bielefeld, Germany), and they were starved for 2 days prior to DNA isolation.

### Sample preparation and DNA isolation

DNA was isolated from four different source samples. Two samples (Fc1 and Fc3) consisted of guts dissected from *F*. *candida* individuals; one sample (Fc4) consisted of whole springtails; one sample (Fc2) consisted of a mixture of whole animals and dissected guts. Dissected guts were rinsed in sterile PBS and whole springtails were rinsed three times in sterile water before processing. After the washing steps, DNA was directly isolated from two of the samples (Fc3 and Fc4) while additional steps were applied to prepare samples Fc1 and Fc2. For these two samples, we separated bacterial cells from *F*. *candida*’s cells by using the method described by Engel *et al*.^22^, with modifications. The samples were crushed in PBS in a 1.5 ml microcentrifuge tube, using a plastic pestle. The samples were then gently vortexed, to encourage separation of cells, before being passed through 20 µm and 8 µm filters in succession. The filtered samples were centrifuged at 10 000 g for 30 min to harvest cells, and the pellet was resuspended in 200 µl TE buffer. For sample Fc2, an additional step with a density gradient was applied. An 80% Percoll solution in 0.15 mol l^−1^ NaCl was prepared. 1 ml of this solution was placed in a 2 ml microcentrifuge tube and spun at 20 000 g for 20 min to create a gradient. The 200 µl of TE buffer containing the cells was gently placed on top of the gradient, and the tube was centrifuged at 400 g for 20 min. Bacterial cells were then visible as a band and were collected using a pipette. The cells were centrifuged at max speed for 5 min and washed with TE buffer to remove residual Percoll solution. DNA was extracted from all samples using the PowerSoil DNA Isolation Kit (MOBIO Laboratories Inc., Carlsbad, CA, USA) and quantified using a Qubit 2.0 (Invitrogen, Carlsbad, CA, USA).

### Library preparation and sequencing

Metagenomic libraries for the four samples were prepared using the TruSeq Nano DNA Library Preparation Kit (Illumina Inc., San Diego, CA, USA) with the following modifications. First, genomic DNA (250 ng) was sheared in a Covaris S2 (Covaris Inc., Woburn, MA, USA) with the following settings: duty cycle 10%, intensity 5.0, bursts per second 200, duration 300 s, mode frequency sweeping, power 23 W, temperature 5.5 °C to 6 °C. Fragmented DNA was cleaned using Agencourt AMPure XP beads (Beckman Coulter Inc., Brea, CA, USA) to remove short fragments. After end repair, cleaning was performed again to select the appropriate library size (180 bp). Then, 3′ end adenylation and adapter ligation were performed, and the ligated fragments were subjected to two rounds of clean-up. PCR was used to enrich the ligated DNA fragments. The PCR program started with 3 min at 98 °C, followed by eight amplification cycles (20 s at 98 °C, 15 s at 60 °C and 30 s at 72 °C) and a final extension step of 5 min at 72 °C. The amplified library was cleaned and its quality was assessed with a Bioanalyzer on a DNA 7 500 chip (Agilent Technologies, Santa Clara, CA, USA). Finally, libraries were equimolarly combined and the concentration of the final pool was checked using a High Sensitivity DNA chip. 10 pmol of barcoded DNA was sequenced on an Illumina HiSeq 2 500 using 125 base, paired end run mode.

### Data analysis

Raw reads of the four samples obtained from the sequencer were trimmed using Trimmomatic version 0.36^85^ to remove adapters and low quality reads, with the following options: ILLUMINACLIP:TruSeq3-PE.fa:2:30:10, LEADING:3, TRAILING:3, SLIDINGWINDOW:4:20, MINLEN:36. Metaphlan2 was used to characterize the taxonomic profile of the metagenome^86^. Bowtie2^87^ was used to create reference genomes for *Folsomia candida* (BioProject accession: PRJNA299291)^10^, *Wolbachia pipientis* (BioProject accession: PRJNA300838)^10^, *Saccharomyces cerevisiae* (Assembly accession: ASM105121v1) and *Homo sapiens* (Assembly accession: GRCh38.p7), and to align and identify reads originating from these organisms in the metagenome. SAMtools was used to remove the reads aligned to the reference genomes of the above mentioned organisms from the metagenome. This program was also used to merge all the four sequencing samples together for comprehensive bioinformatic analysis^88^. Only paired ends were extracted with Bedtools^89^. FastQC^90^ was used to check the quality of the reads at different processing stages. Assembly was done using SPAdes version 3.9.0 with the (–meta) setting for metagenomic and k-mer values 21, 41, 65, 75, 87, 91, 95. This range of K-mer was found to give the best assembly result^91^. The quality of contigs was checked with Quast 4.2^92^. Prodigal (version 2.6.3) was used for genes prediction with the option -m -p meta for predicting metagenomic genes with no gaps^93^. Taxonomic assignment was done using Metaphlan2. The predicted proteins were uploaded to GhostKOALA webservice for KEGG assignment^94^. For functional annotation, blastp was performed against the Swiss-Prot, refseq and NR databases, with a threshold e-value of 1e-6. InterProScan5 was used with the addition of panther database to identify protein domains using HMM model^95^. Blast2GO was used to integrate the blastp and interproscan results for further improving functional annotation^96^. HMMER version 3.0 was used with CAZy database (version 6) using HMM model to identify carbohydrate-active genes^97^. These genes were subjected to filtering using an e-value threshold of 1e-5 for alignments over 80aa, and a threshold of 1e-3 for shorter alignments. The CARD database was used to identify resistance genes^21^. All the amino acid sequences of anti-resistance proteins were merged and subjected to blastp with a threshold e-value of 1e-6. All the sequences with more than 60% identity with their top blast hit were collected. Descriptions of the ARO terms was obtained from the online database (https://card.mcmaster.ca/). The KEGG, Pfam and NR databases were used to confirm the accuracy of the functional annotations obtained with CAZY and CARD. Secondary metabolite biosynthetic gene clusters were identified for contigs larger than 3 000 bp using the antiSMASH2 program^98^. To identify homologies and orthologies between the genome of *F*. *candida* and the metagenome, a reciprocal blast was performed. The metagenomic protein sequences were blasted against the host proteins, and vice versa. Sequences that were top hits of each other were extracted using a homemade script, and those matching *F*. *candida*’s foreign genes were identified^10^. For a detailed explanation of the methods used to identify the foreign genes within the genome of the springtail, we refer to the publication from Faddeeva-Vakhrusheva *et al*.^10^. Phyre2 was used to predict the structure of the protein of the best reciprocal blast hits^99^.

### Data deposition

The raw sequencing data was deposited in NCBI’s Sequence Read Archive (SRA) under accesison number SRP149127. The Whole Genome Shotgun (WGS) project was deposited at DDBJ/ENA/GenBank under accession number QIRE00000000. The version described in this paper is version QIRE01000000.

## Supplementary information

Supplementary Information File

Supplementary Figures and Table

Supplementary Dataset 1

Supplementary Dataset 2

Supplementary Dataset 3

Supplementary Dataset 4

Supplementary Dataset 5
