# Supplementary material for: Antimicrobial activity and carbohydrate metabolism in the bacterial metagenome of the soil-living invertebrate Folsomia candida
Source: Sci Rep. 2019 May 13;9:7308. doi: 10.1038/s41598-019-43828-w (PMC6513849; doi:10.1038/s41598-019-43828-w)
Supplement: Supplementary file 1 — Supplementary Information File [file 41598_2019_43828_MOESM1_ESM.docx]

**Antimicrobial activity and carbohydrate metabolism in the bacterial metagenome of the soil-living invertebrate *Folsomia candida***

Valeria Agamennone, Giang LeNgoc, Nico M. van Straalen, Abraham Brouwer, Dick Roelofs

**Supplementary Files**

**Supplementary Figure 1**. Contig length distribution.

**Supplementary Figure 2**. Identified taxonomic groups at the phylum, class and genus level.

**Supplementary Figure 3**. Diagram of the pathways involved in starch and sucrose metabolism. Pink boxes indicate the genes identified in the microbiome. The pathway map was obtained from the KEGG database ^100^.

**Supplementary Figure 4**. Predicted protein structures of the top three reciprocal blast hits between the metagenome and the genome of *F. candida*, corresponding to a glycosidase (A), an arabinosidase (B), an isocitrate lyase (C). The predicted structures of the microbial genes are on the left, the predicted proteins of the springtail are on the right.

**Supplementary Table 1**. Summary of antiSMASH results.

**Supplementary File 1**. List of all the predicted genes with the corresponding taxonomies (based on MetaPhlan) and functional annotations (based on NCBI protein database).

**Supplementary File 2**. Complete list of the 2 004 genes predicted to code for enzymes involved in carbohydrate metabolism. For each gene, the following information is given: Carbohydrate Active EnZyme (CAZy) hit, Pfam (protein family), KEGG and NR (non-redundant database) annotations, taxonomic assignment, and whether the gene has a reciprocal blast hit against one of the horizontally transferred genes in *F. candida*’s genome. In this latter case, further information on the hit is given as follows: percentage identity between the metagenomic read S and the *F. candida* contig F over the length of the coverage area; length of the coverage area; e-value of the match; bit score of the match.

**Supplementary File 3**. Complete list of antiSMASH results. For each of the 166 contigs with a hit in the antiSMASH database, the following information is given: the type of cluster, the specific location on the contig, the most similar known cluster (when available) and the BGC (Biosynthetic Gene Cluster) identity in the MIBiG (Minimum Information about a Biosynthetic Gene cluster) repository.

**Supplementary File 4**. Complete list of the predicted genes with a hit to antibiotic resistance in the Comprehensive Antibiotic Resistance Database (CARD)^21^. For each gene, the ARO (Antibiotic Resistance Ontology) accession number and the CARD classification and category are indicated.

**Supplementary File 5**. List of all best reciprocal hits between *Folsomia candida*’s genome and metagenome that are also predicted foreign genes (HGT), including their taxonomic and functional annotation. For each match between a metagenomic sequence (S) and a *F. candida*’s contig (F) the following information is given for the reciprocal comparison (S versus F and F versus S): percentage identity between the metagenomic read and the *F. candida* contig over the length of the coverage area; length of the coverage area; e-value of the match; bit score of the match.
