# Supplementary material for: Antimicrobial activity and carbohydrate metabolism in the bacterial metagenome of the soil-living invertebrate Folsomia candida
Source: Sci Rep. 2019 May 13;9:7308. doi: 10.1038/s41598-019-43828-w (PMC6513849; doi:10.1038/s41598-019-43828-w)
Supplement: Supplementary file 2 — Supplementary Figures and Table [file 41598_2019_43828_MOESM2_ESM.pdf]

Antimicrobial activity and carbohydrate metabolism in the bacterial metagenome of the soil-living invertebrate *Folsomia candida*

Valeria Agamennone, Giang LeNgoc, Nico M. van Straalen, Abraham Brouwer, Dick Roelofs

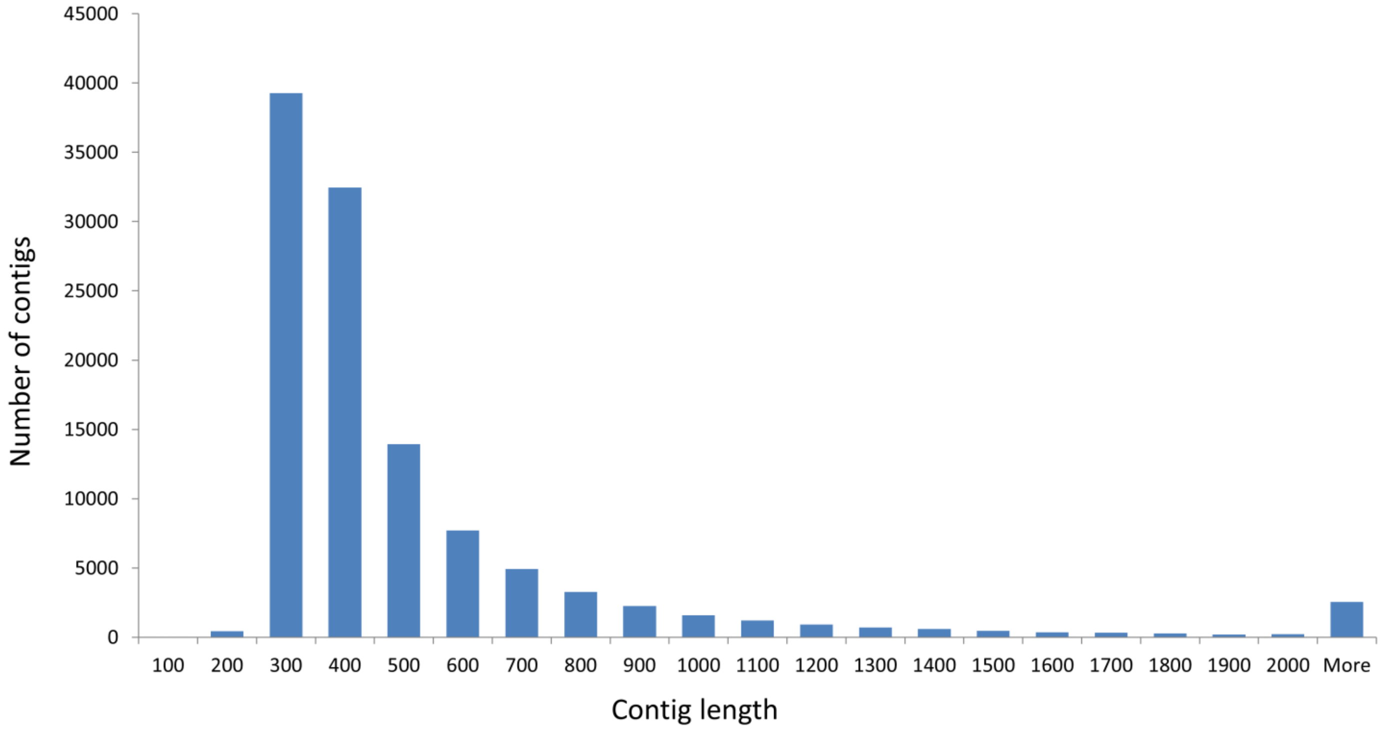

Supplementary Figure 1. Contig length distribution.

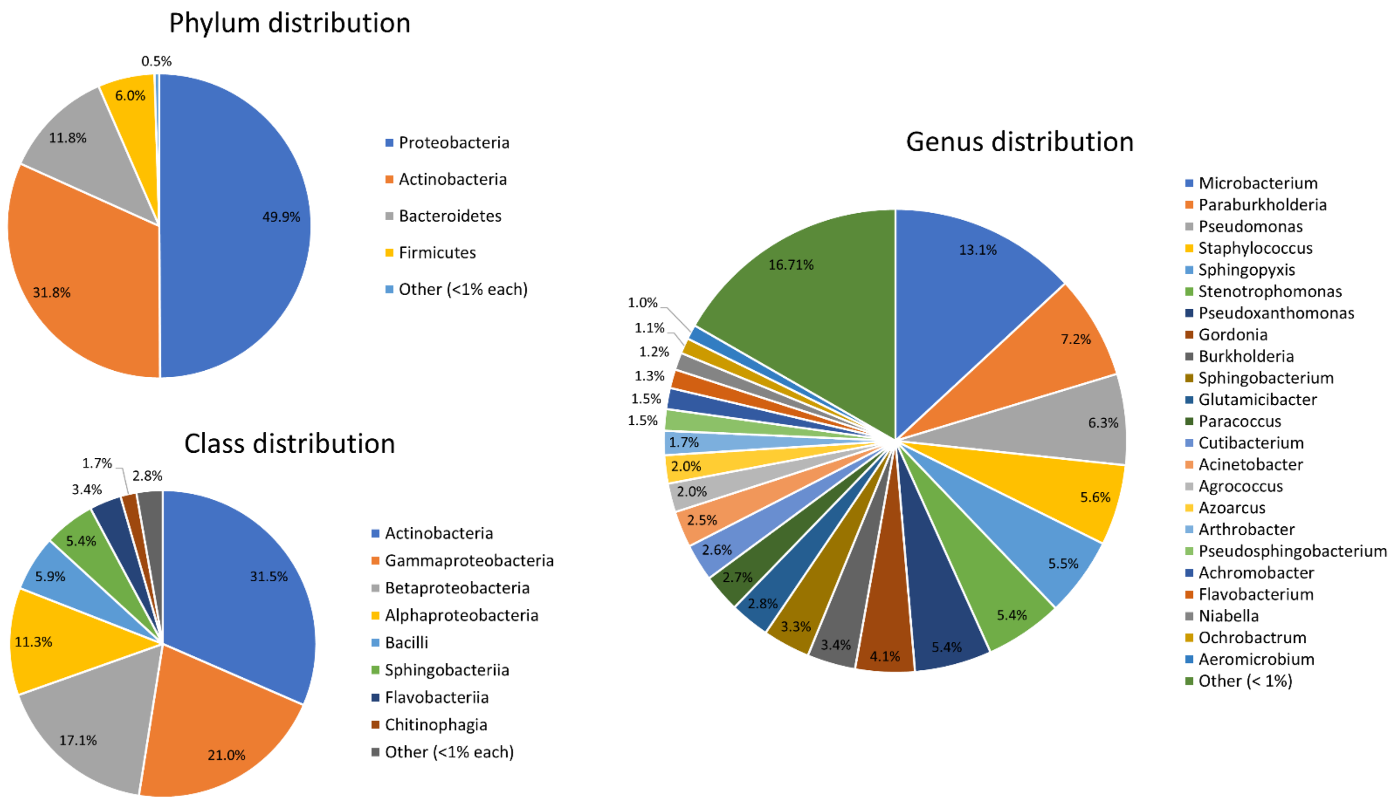

Supplementary Figure 2. Identified taxonomic groups at the phylum, class and genus level.

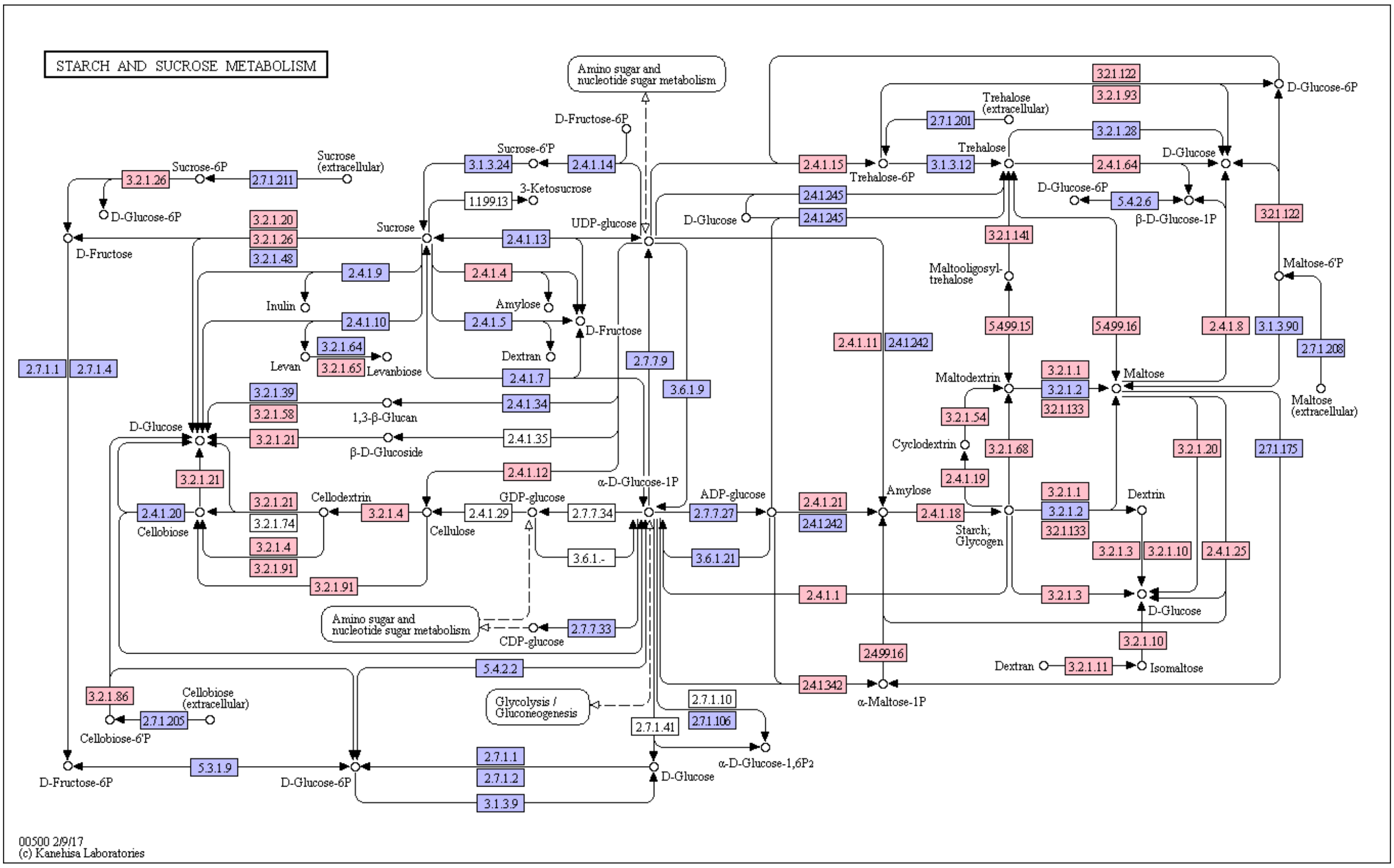

Supplementary Figure 3. Diagram of the pathways involved in starch and sucrose metabolism. Pink boxes indicate the genes identified in the microbiome. The pathway map was obtained from the KEGG database [100].

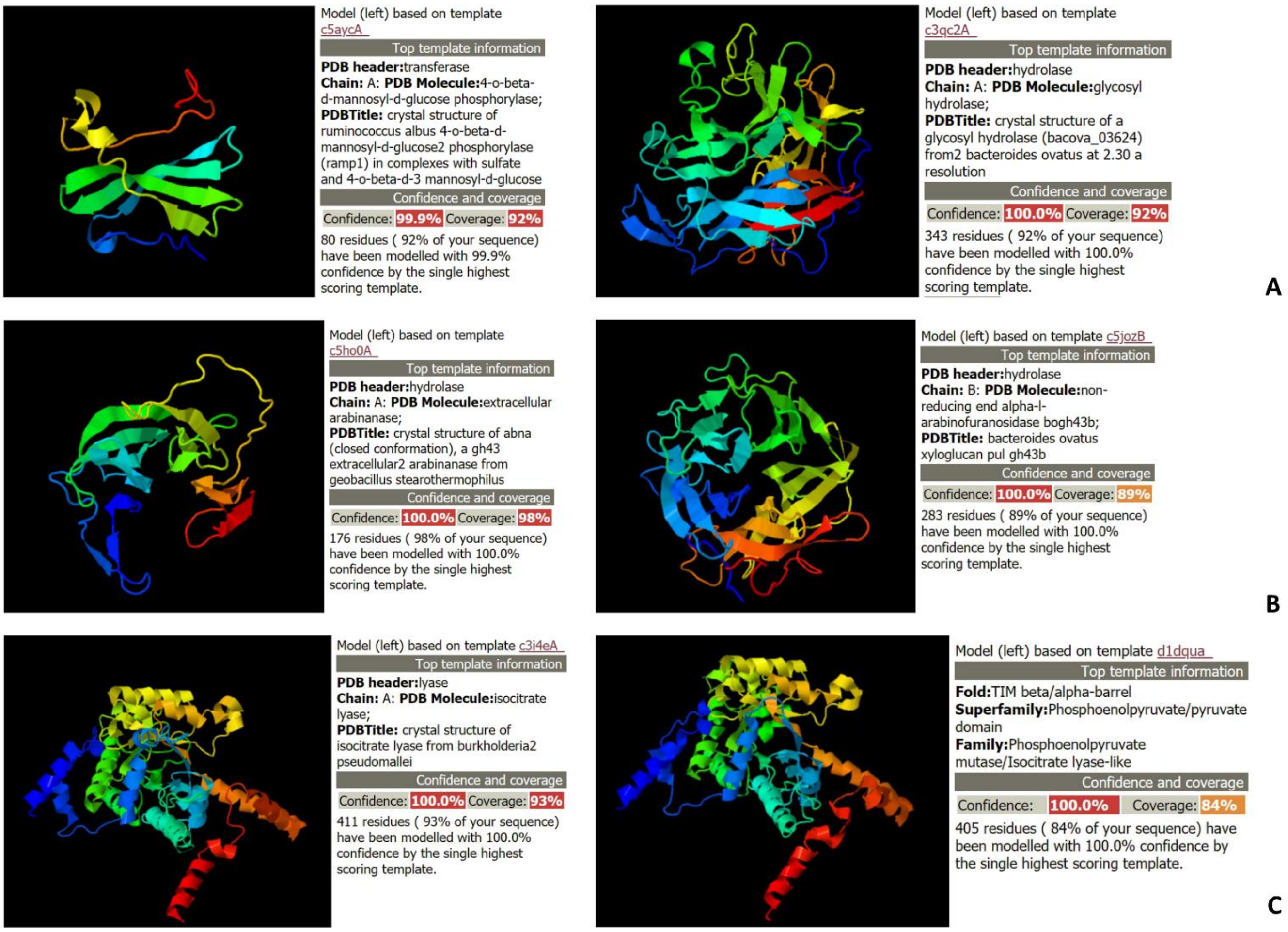

Supplementary Figure 4. Predicted protein structures of the top three reciprocal blast hits between the metagenome and the genome of *F. candida*, corresponding to a glycosidase (A), an arabinosidase (B), an isocitrate lyase (C). The predicted structures of the microbial genes are on the left, the predicted proteins of the springtail are on the right.

Supplementary Table 1. Summary of antiSMASH results

**Cluster type**	**Number of contigs**
Arylpolyene	1
Bacteriocin	4
Cf_fatty_acid	14
Cf_fatty_acid -Cf_saccharide	1
Cf_putative	96
Cf_saccharide	18
Cf_saccharide-Cf_fatty_acid	1
Ectoine	1
Hserlactone	3
Nrps	13
Nrps-Arylpolyene	1
Other	2
Siderophore	2
T1pks	1
T1pks-Nrps	1
T3pks	1
T3pks-Cf_saccharide	1
Terpene	5
**TOTAL**	**166**

For each type of secondary metabolite cluster, the number of contigs in *F. candida*'s metagenome in which the cluster was detected is indicated. Cf indicates a putative cluster identified with the ClusterFinder algorithm. Pks = polyketide synthase. Nrps = non-ribosomal peptide synthetase. The complete output of the antiSMASH analysis is given in Supplementary File 3.
